# Early smoking lead to worse prognosis of COPD patients: a real world study

**DOI:** 10.1186/s12931-024-02760-y

**Published:** 2024-03-25

**Authors:** Jiankang Wu, Weiwei Meng, Yiming Ma, Zhiqi Zhao, Ruoyan Xiong, Jiayu Wang, Rui Zhao, Huihui Zeng, Yan Chen

**Affiliations:** 1 Department of Pulmonary and Critical Care Medicine, The Second Xiangya Hospital, Central South University, 139 Middle Renmin Road, Changsha, 410011 Hunan China; 2 Research Unit of Respiratory Disease, Central South University, Changsha, 410011 Hunan China; 3 Clinical Medical Research Center for Pulmonary and Critical Care Medicine in Hunan Province, Changsha, 410011 China; 4 Diagnosis and Treatment Center of Respiratory Disease, Central South University, Changsha, 410011 Hunan China

**Keywords:** Early smoking, COPD, Clinical outcomes, Prognosis

## Abstract

**Background:**

Smoking remains a major risk factor for the development and progression of chronic obstructive pulmonary disease (COPD). Due to the adolescent smoking associated with worse health state, the age, at which an individual started smoking, might play a key role in shaping the trajectory of COPD development and the severity.

**Methods:**

We conducted an observational study from September 2016 through January 2023 of eligible patients hospitalized with COPD. Patients who started smoking during the alveolar development stage (ADS, smoking initiation ≤ 24 years old) were defined as early smoking patients, and patients who started smoking after ADS (smoking initiation > 24 years old) were defined as late smoking patients. We collected demographic and clinical data characterizing the patients and documented their condition from hospital discharge to follow-up. The primary endpoints were short-term (within one year), 3-year, and long-term (beyond 3 years) all-cause mortality after discharge.

**Results:**

Among 697 COPD patients, early smoking patients had a lower smoking cessation rate (*P* < 0.001) and a higher smoking index (*P* < 0.001) than late smoking patients. Although adjusted smoking index, early smoking patients still had poorer lung function (*P* = 0.023), thicker left ventricular diameters (*P* = 0.003), higher frequency of triple therapy use during stable stage (*P* = 0.049), and more acute exacerbations in the past year before enrollment (*P* < 0.05). Survival analysis showed that they had a higher risk of death after discharge within three years (*P* = 0.004) and beyond three years (*P* < 0.001). Furthermore, even in early smoking COPD patients who quit smoking after adjusting the smoking index had poorer lung function (*P* < 0.05) and thicker left ventricular diameters (*P* = 0.003), and survival analysis also showed that they had a higher long-term mortality rate (*P* = 0.010) and shorter survival time (*P* = 0.0128).

**Conclusion:**

Early smoking COPD patients exhibited multiple adverse clinical outcomes, including heavy cigarette addiction, compromised pulmonary function, augmented left ventricular diameter, and elevated mortality risk. Additional, smoking cessation could not bring enough improvement of health state in early smoking COPD patients as late smoking COPD patients. Consequently, early intervention and specialized cessation approaches for younger smokers are of paramount importance in this context.

**Supplementary Information:**

The online version contains supplementary material available at 10.1186/s12931-024-02760-y.

## Introduction

Chronic obstructive pulmonary disease (COPD) is a common, preventable, and treatable airway disease with progressive airflow limitation. It is associated with high morbidity and mortality, making it a global public health concern [1, 2]. Two Global Burden of Disease reports indicate that COPD is the 8th leading cause of disability in the global population and the 3rd leading cause of death in the world [3, 4]. The findings from the 2019 China COPD Epidemiology Survey reveal a staggering statistic: close to 100 million individuals in China are grappling with COPD, with a prevalence rate of 13.7% among those aged 40 and above [5]. Smoking remains the predominant risk factor to COPD, accounting for approximately 80–90% of diagnosed cases [6]. A Chinese cohort study underscores the gravity of the situation, reporting one million direct cigarette-related deaths in 2010, making the implementation of proactive interventions and preventive measures crucial [7].

In addition, regarding the adolescent population, studies have shown a worrying trend. Gender-standardized smoking prevalence among adolescents aged 15–24 years has increased by 50.6% from 8.3% in 2003 to 12.5% in 2013 [7]. A 2020 study highlights a troubling change in China’s smoking population, which is a significant decrease in the age at which individuals start smoking [8]. Adolescent smoking, with the introduction of toxins and harmful substances during this sensitive period, may cause irreversible damage to physical and psychological development, leading to even worse outcome [9–11]. Rather than adolescence, alveolar development stage (ADS), a special stage for alveolar development, is the process of increasing the number of alveoli and maturing during the period from birth until approximately 24 years of age [12–14]. Due to the possible irreversible damage, smoking during ADS may not only lead to a decrease in the number of alveoli, but may also cause long-term abnormalities in the structure and function of the lungs. These developmental abnormalities are one of the six major causes of COPD listed in the Lancet Commission Report [15]. While much is understood about the overall relationship between smoking and COPD, there remains a significant knowledge gap between the early smoking initiation during adolescence and the subsequent development of COPD. Bridging this gap is essential for targeted prevention strategies.

Quitting smoking provides numerous health benefits, many of which become evident shortly after cessation. Within just 20 minutes of quitting, heart rate and blood pressure begin to decline and substantial improvements occur in the circulatory system and lung function within 2 to 12 weeks, such as a reduction in coughing and shortness of breath within 1 to 9 months. Importantly, after a year of quitting, the risk of coronary heart disease is roughly halved and the risk of smoking-related mortality gradually diminishes with more than a decade of abstinence [16–21]. Despite the well-documented advantages of quitting smoking, a substantial percentage of people continue to smoke, often grappling with addiction, withdrawal symptoms, and concerns about weight gain post-cessation [22]. However, further research is needed to define whether the health benefits of smoking cessation for adolescent smokers are “sufficient for physiologic markers, rebound in lung function, and improved quality of life”. Considering the high prevalence of tobacco use in adolescents and its associated health risks, research in this area is needed. Through discussing the interactions between age of smoking initiation and COPD developmental trajectories, this study aims to reveal potential adverse impact of early smoking initiation patterns in the severity and prognosis of COPD.

## Methods

### Patients

**Fig. 1:**
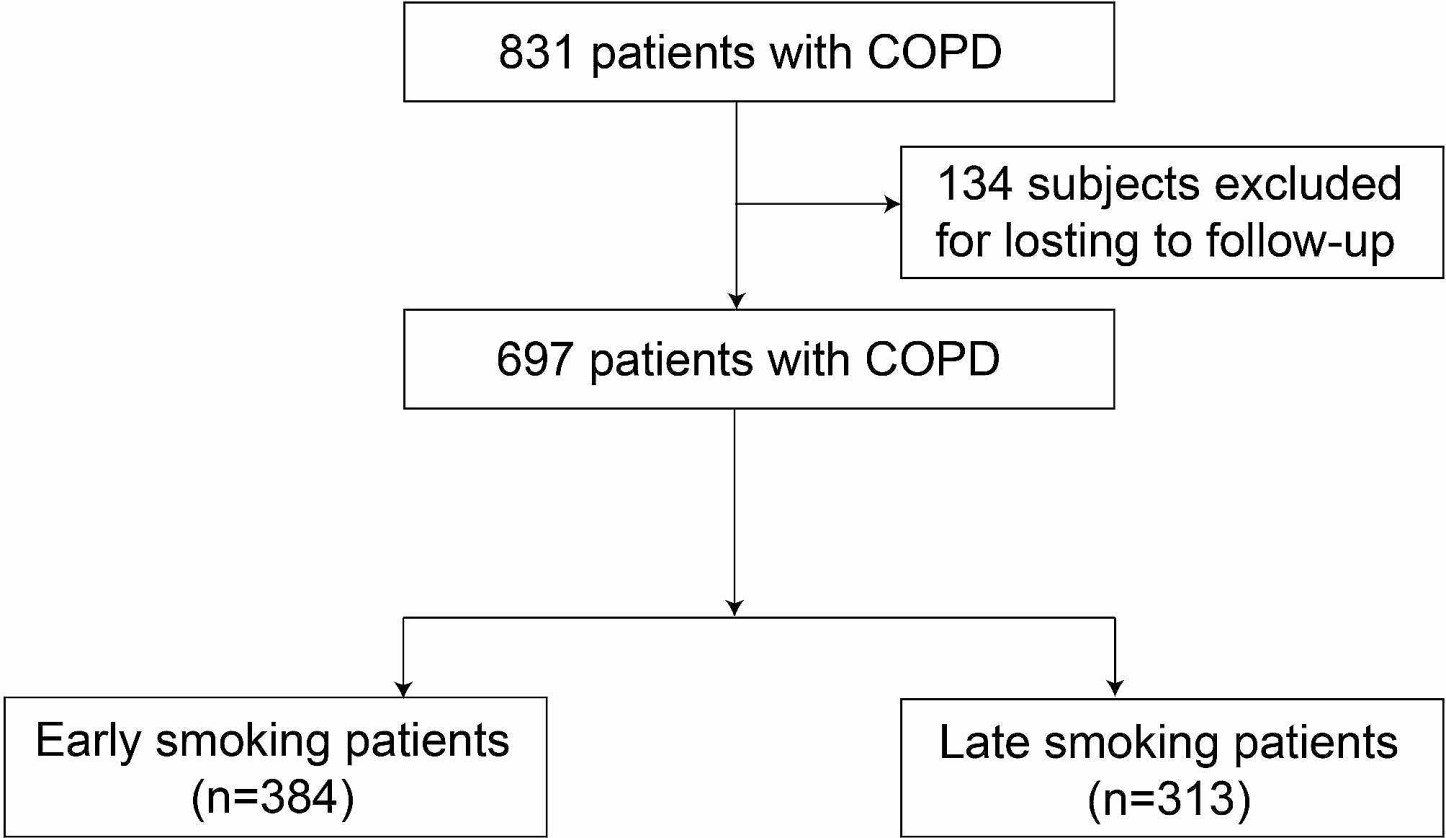
The flow diagram of eligible COPD patients

This study recruited a cohort of eligible hospitalized patients with COPD from September 2016 to July 2020 at the Department of Pulmonary and Critical Care Medicine, the Second Xiangya Hospital of Central South University. Inclusive criteria included were as follows: (1) patients diagnosed with COPD according to the definitons of 2016 GOLD guidelines; (2) a history of smoking; (3) signed informed consent. Patients who died during hospitalization or refused to co-operate with interviews were excluded from the study (Fig. 1).

## Data collection

The demographic and clinical data of patients were collected, including gender, age, BMI, smoking status, the severity of COPD evaluated according to GOLD guidelines, comorbidities, cardiac function, routine blood examination, arterial blood gas analysis, and spirometry tests. Dyspnea and respiratory symptoms were evaluated by the modified Medical Research Council (mMRC) Dyspnea Scale and COPD Assessment Test (CAT).

### Follow-up

The enrolled patients were followed up by telephone after discharge. Information collected during the first year of follow-up included the frequency of exacerbations and the frequency of readmissions due to Acute Exacerbation of Chronic Obstructive Pulmonary Disease (AECOPD) in previous year and whether they died, as well as the time of death. Subsequent annual follow-up only inquired survival status. Survival time was recorded until either the patient’s death or December 2022 for survival analysis purposes. Those who died within one year after discharge were included in the analysis of short-term mortality, while those who died during the whole follow up were included in the analysis of long-term mortality.

### Statistical analysis

SPSS 26.0 (IBM, New York, USA) software was used for all statistical analysis. Continuous variables were presented as mean and SD, and analyzed by t test. Non-parametric data were presented as median and IQR, and analyzed by Mann-Whitney test. For categorical variables, numbers and percentages were used, and chi square test was used for univariate analysis. To correct for differences in smoking indices between the two patient groups, an analytic sample was generated using a propensity score-based matching method. Propensity score matching was performed in a 1:1 ratio by nearest neighbor matching. Propensity modeling was appropriate by examining the balance of covariates before and after adjusting. After adjusted the smoking index, both groups were analyzed for 1-year, 3-year and long-term survival. Kaplan-Meier survival curves was used for univariate survival analysis. *P* < 0.05 was considered significant. Graphs were jointly completed by SPSS 26.0 and GraphPad Prism 9.

## Results

Our study underwent 1-year, 3-year and long-term follow-up with a median follow-up time of 41 months and a maximum follow-up time of 74 months. 697 patients met the inclusion criteria and 134 patients were lost to follow-up. A total of 194 (27.8%) patients died during the entire follow-up. Of these, 75 (10.7%) patients died within one year. The number of early smoking patients was 384 patients, and late smoking patients was 313. After adjusting according to the smoking index, there were 149 patients in each group. Among the patients who quit smoking, there were 206 early smoking patients and 253 late smoking patients. After adjusting for smoking index, there were 104 patients in each group.

### Demographic characteristics

The overall median age for the population was 68 years with 97.6% being male. The median age of smoking initiation in early smoking patients was 18 years and 98.7% are male. The median age of late smoking COPD patients was 33 years and 96.2% were male. Comparing the baseline data at admission between the two groups (Supplementary Table 1), early smoking COPD patients were currently younger than late smoking COPD patients (66 years old vs. 72 year old, *P* < 0.001). However, we could not find any other differences in the other demographic data between the two groups.

### Early smoking leading to severer cigarette addiction

**Fig. 2:**
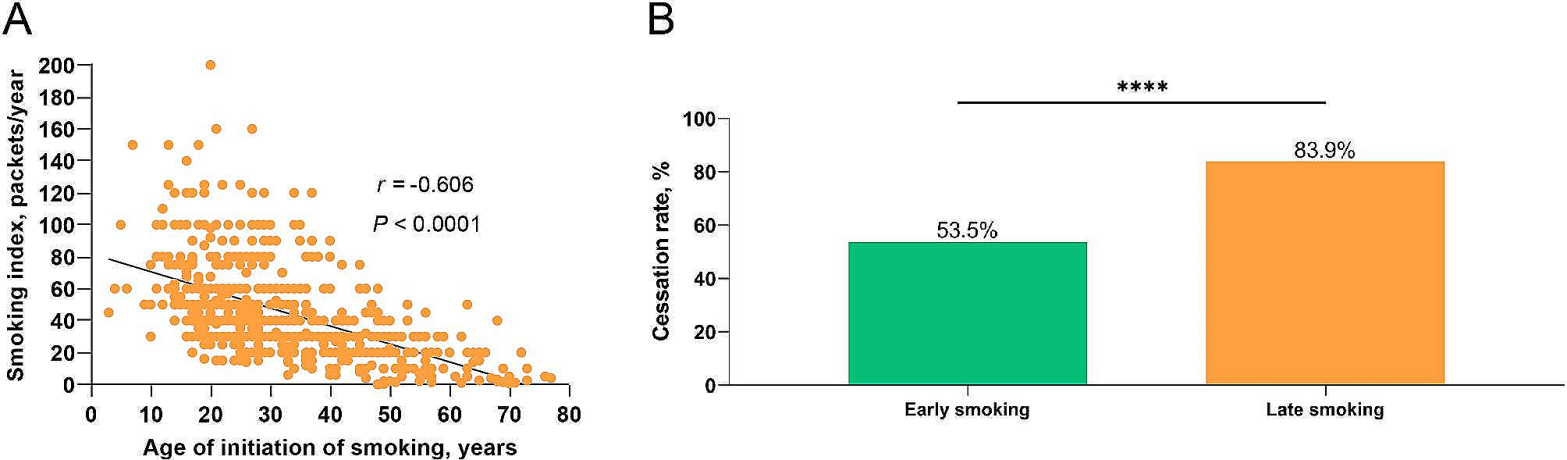
Correlation analysis between age of smoking initiation and smoking index (**A**). Comparison of smoking cessation rates in early smoking or late smoking COPD patients (**B**)

Compared to late smoking patients, the early smoking COPD population had longer smoking histories and higher smoking indices (50 vs. 30 packets/year, *P* < 0.0001), despite early smoking COPD population were at younger age (Supplementary Table 1). In addition, early smoking COPD patients had lower rates of smoking cessation than late smoking COPD patients. (53.5% vs. 83.9%, *P* < 0.0001, Fig. 2), suggesting that early smoking might lead to heavier smoking burden and severer cigarette addiction.

### Early smoking associated with worse condition at the beginning of AE

In the whole hospitalized population for AECOPD population, early smoking patients were found to have poorer lung function (FEV1/FVC, 35.95% vs. 38.61%, *P* = 0.011), higher frequency of adverse events in the past 12 months before admission (2 vs. 1, *P* = 0.031), and higher PaCO_2_ (51 vs. 49 mm/Hg, *P* = 0.023, Supplementary Table 1) at the beginning of admission. We performed propensity score matching to account for the effect of smoking index factors and found that adjusted early smoking COPD cohort had poorer lung function (FEV1/FVC, 34% vs. 38.89%, *P* = 0.023), a higher frequency of adverse events in the past 12 months before hospitalization (2 vs. 1, *P* = 0.011) and a greater reliance on triple therapy during stabilization (38.9% vs. 27.5%, *P* = 0.049, Table 1).

**Table 1:** Baseline characteristics (after adjusting smoking index)

Variables		Early smoking (*n* = 149)		Late smoking (*n* = 149)		*P*-value
Age, years		69(65.5,78)		70(67, 79)		0.035
Male, %		148(99.3%)		146(98.0%)		0.314
BMI, kg/m^2^		21.63(18.72,24.31)		21.16(18.67,24.45)		0.736
Smoking index, packets/year		40(30,56.5)		40(30, 60)		0.991
Spirometry(post-bronchodilation)						
FEV1% predicted		29(21,39.6)		32.1(23,45)		0.116
FEV1/FVC, %		34(27.8,43.24)		38.89(31.1,46.85)		0.023
mMRC		3(2,4)		3(2,4)		0.159
CAT		23(18,28)		24.5(19,29)		0.429
Frequency of AEs in the last 12 months, times		2(1,2)		1(1,3)		0.011
Frequency of admission for AECOPD in the last 12 months, times		2(1,2)		1(1,2)		0.031
Laboratory investigations on admission						
WBC count, x 10^9^/L		7.37(5.93,10.25)		7.23(5.97,9.73)		0.746
Neutrophil count, x 10^9^/L		5.44(4.02,7.66)		5.05(4.02,8.43)		0.478
Eosinophil count, x 10^9^/L		0.14(0.06,0.25)		0.13(0.05,0.23)		0.127
CRP, mg/l		6.45(2.97,29.63)		11.3(4.45,32.77)		0.050
PCT, mg/l		0.05(0.05, 0.12)		0.1(0.05, 0.14)		0.264
BNP, pg/ml		135(50, 930)		133(50, 560)		0.917
PaCO_2_, mm/Hg		50(43,61.3)		50(42,62)		0.331
PaO_2_, mm/Hg		68(56,80)		64(53,76)		0.489
SaO_2_, %		93 (88,96)		92 (87,95)		0.704
ICS therapy during stable stage, %		81.2		77.9		0.474
Triple therapy during stable stage, %		38.3		27.5		0.049

**Notes**: Date are presented as median(IQR) or n(%). FVC: forced vital capacity; FEV1: forced expiratory volume in 1 s; mMRC: modified Medical Research Council; CAT: COPD Assessment Test; TB: tuberculosis; AE: Acute Exacerbation; AECOPD: Acute Exacerbation of Chronic Obstructive Pulmonary Disease; CRP:C-reactive protein; PCT: Procalcitonin; BNP: brain natriuretic peptide; PaO2: partial pressure of oxygen in artery; PaCO2: partial pressure of carbon dioxide in arterial blood; SaO2: oxygen saturation in arterial blood; ICS: inhaled corticosteroids

### Early smoking and comorbidity

In terms of comorbidities, there was no significant difference in the prevalence of comorbidities such as coronary artery disease, hypertension, diabetes, pneumonia, bronchiectasis, respiratory failure, prior pulmonary TB, and cor pulmonale between the two groups of patients (Table 2). However, the ultrasound results showed that left ventricular diameters were thicker in early smoking COPD patients than late smoking COPD patients (44 vs. 42 mm, *P* = 0.016, Supplementary Table 2), and after adjusted the differences seemed to be more significant (44 vs. 42 mm, *P* = 0.003, Table 2) .

**Table 2:** The effect of early or late smoking on comorbidities and left heart function in COPD patients (after adjusting smoking index)

Variables		Early smoking (*n* = 149)		Late smoking (*n* = 149)		*P*-value
Echocardiography						
LVD, mm		44(40,47)		42(38,44)		0.003
LAS, mm		30(26.25,33)		30(27,33)		0.589
RVD, mm		29(26,32)		29(27,32)		0.899
RAS, mm		29(26,32)		29(27,32)		0.744
AO, mm		29(28,32)		30(27,32)		0.559
PA, mm		22(20,24)		22(20,25)		0.852
PA/AO		0.75(0.69,0.83)		0.75(0.69,0.88)		0.948
LVSD, %		9(8,10)		9(8,10)		0.806
LVPWD, mm		9(8,10)		9(8,10)		0.497
PAV, cm/s		90(80,100)		90(80,100)		0.235
EF, %		61(60,64)		60(60,62)		0.601
FS, %		32(30,34)		32(30,34)		0.565
Comorbidities and complications						
Coronary heart disease, %		12.8		18.8		0.153
Hypertension, %		34.2		38.3		0.470
Diabetes, %		12.1		9.4		0.454
Pneumonia, %		14.8		19.5		0.282
Bronchiectasis, %		8.7		14.8		0.105
Respiratory failure, %		43.6		44.6		0.866
Prior pulmonary TB, %		25.5		30.6		0.328
Cor pulmonale, %		25.5		24.8		0.894

**Notes**: LVD: Left Ventricular Diameter; LAS: Left Ventricular Diameter; RVD: Reft Ventricular Diameter; RAS: Left Ventricular Diameter; AO: Aortic Diameter; PA: Pulmonary Artery Diameter; LVSD: Left Ventricular Systolic Dysfunction; LVPWD: Left Ventricular Posterior Wall Diameter; PAV: Pulmonary Artery Velocity; EF: Ejection Fraction; FS: Fractional Shortening

### Early smoking patients and all-cause mortality

**Fig. 3:**
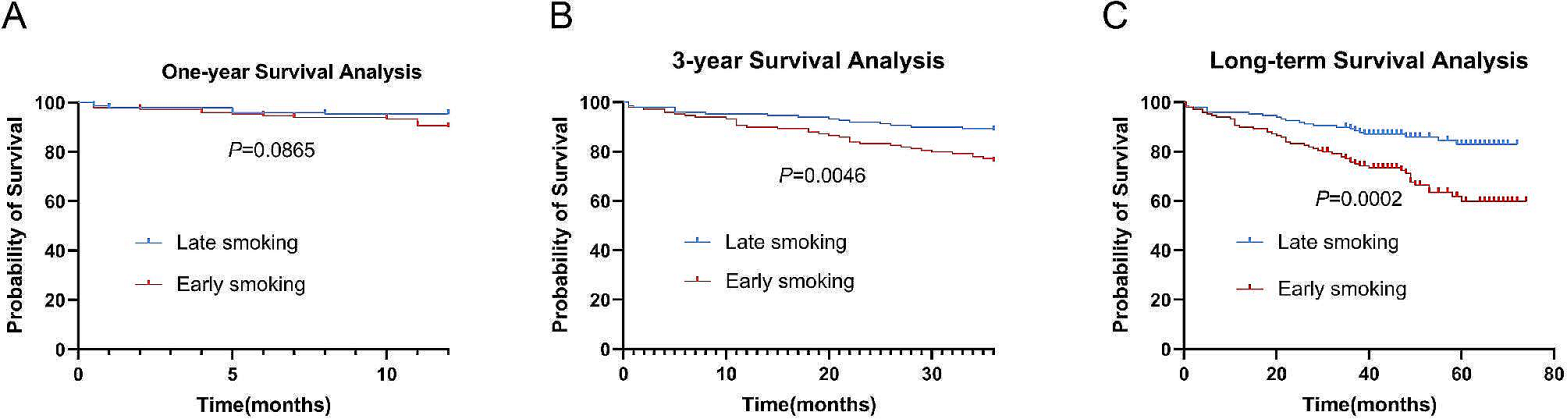
Kaplan-Meyer survival curves for the effect of early or late smoking on one-year (**A**), three-year (**B**), and long-term (**C**) mortality in paired COPD patients

Although the direct comparison could not find the differences in all-cause mortality between early smoking and late smoking COPD patients (Supplementary Table 3), after adjusting the possible bias factor, smoking index, exhibited elevated three-year (24.2% vs. 11.4%, *P* = 0.004, Table 3) and long-term mortality rates (32.9% vs. 15.4%, *P* < 0.001, Table 3) along with reduced survival durations in early smoking COPD patients (Fig. 3).

**Table 3:** The effect of early or late smoking on prognosis in COPD patients (after adjusting smoking index)

Variables	Early smoking (*n* = 149)	Late smoking (*n* = 149)	*P*-value
Number of mild AEs one year after discharge, times	0(0,0)	0(0,0)	0.904
Number of AECOPDs one year after discharge, times	1(0,2)	1(0,2)	0.660
Incidence of death within one year after discharge(%)	10.1	4.7	0.076
Incidence of death within 3 years after discharge(%)	24.2	11.4	0.004
Incidence of death more than 3 years after discharge(%)	32.9	15.4	< 0.001

**Notes**: AE: Acute Exacerbation; AECOPD: Acute Exacerbation of Chronic Obstructive Pulmonary Disease

### Early smoking leads to poorer smoking cessation outcomes

**Fig. 4:**
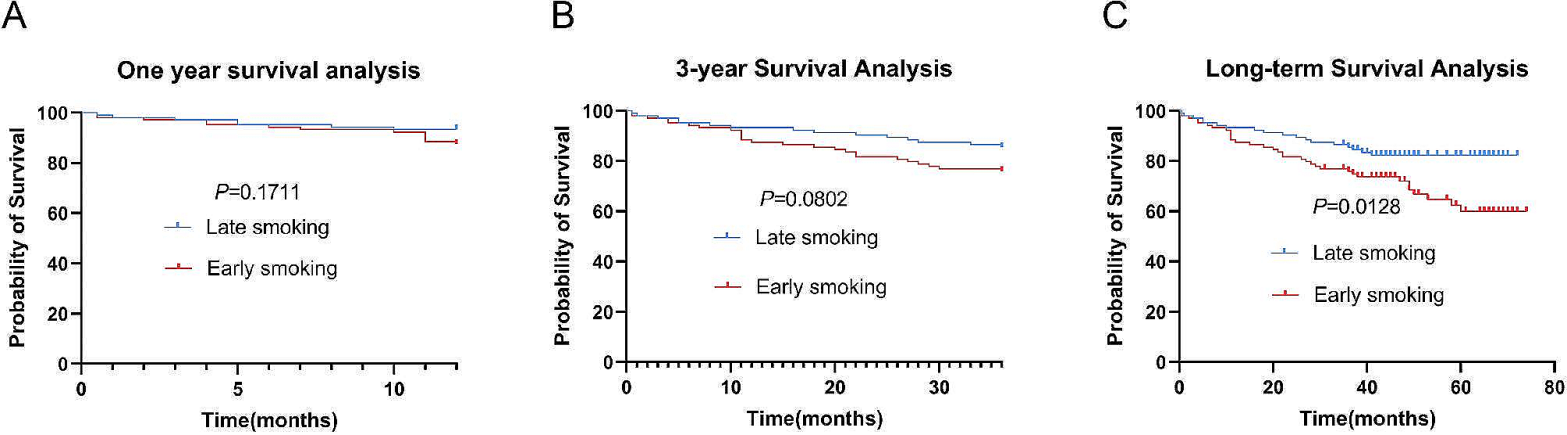
Kaplan-Meyer survival curves for the effect of smoking cessation on one-year (**A**), three-year (**B**), and long-term (**C**) mortality in paired patients who were early or late smoking COPD patients

In the entire cohort of COPD patients who ceased smoking, early smoking patients were found to have poorer lung function (FEV1% predicted: 27.9% vs. 32%, *P* = 0.007; FEV1/FVC: 35% vs. 38%, *P* = 0.017, Supplementary Table 4), and thicker left ventricular diameters than late smoking patients (44 vs. 42 mm, *P* = 0.025, Supplementary Table 5). We performed propensity score matching to account for the effect of smoking index factors and found that poorer lung function (FEV1% predicted: 26% vs. 31.1%, *P* = 0.013; FEV1/FVC: 34.1% vs. 38%, *P* = 0.010, Table 4), and thicker left ventricular diameters (44 vs. 42 mm, *P* = 0.003, Table 5), again survival analysis revealed higher long-term mortality (32.9% vs. 15.4%, *P* = 0.010, Table 6) and shorter survival time (*P* = 0.0128, Fig. 4) in early smoking patients, rather than late smoking patients.

**Table 4:** Early smoking impaired smoking cessation (after adjusting smoking index)

Variables	Early smoking (*n* = 104)	Late smoking (*n* = 104)	*P*-value
Age, years	68(61, 71)	69(65,73)	0.035
Male, %	99	98.1	0.561
BMI, kg/m^2^	22.08(19.36,24.15)	21.21(18.70,24.50)	0.451
Smoking index ,packets/year	40(33,60)	40(33,60)	0.999
Duration of smoking cessation, years	9(3,13)	10(3, 19)	0.787
Spirometry(post-bronchodilation)			
FEV1% predicted	26(19.9,37)	31.1(21,42.3)	0.013
FEV1/FVC, %	34.1(27.3,42.2)	38(30,46.4)	0.010
mMRC	3(2,4)	3(2,4)	0.212
CAT	23(16,27)	28.5(25,34)	0.252
Frequency of AEs in the last 12 months, times	1(1,3)	2(1,3)	0.011
Frequency of admission for AECOPD in the last 12 months, times	1(1,2)	2(1,3)	0.087
Laboratory investigations on admission			
WBC count, x 10^9^/L	7.51(6.3,10.4)	7.18(5.89,9.58)	0.428
Neutrophil count, x 10^9^/L	5.31(4.25,8.36)	5.52(4.02,7.46)	0.098
Eosinophil count, x 10^9^/L	0.14(0.06,0.24)	0.11(0.03,0.23)	0.046
CRP, mg/l	13(3.92,32.5)	9.77(3.89,21.65)	0.861
PCT, mg/l	0.08(0.05, 0.14)	0.08(0.05, 0.15)	0.957
BNP, pg/ml	120(50, 490)	117(50, 555)	0.685
PaCO_2_, mm/Hg	50(43,63)	50(43,62.8)	0.638
PaO_2_, mm/Hg	71(57,85)	65(53,79)	0.351
SaO_2_, %	924(88,96)	92 (87,96)	0.542
ICS therapy during stable stage, %	75	75	1
Triple therapy during stable stage, %	30.8	24	0.277

**Notes**: Date are presented as median(IQR) or n(%). FVC: forced vital capacity; FEV1: forced expiratory volume in 1 s; mMRC: modified Medical Research Council; CAT: COPD Assessment Test; TB: tuberculosis; AE: Acute Exacerbation; AECOPD: Acute Exacerbation of Chronic Obstructive Pulmonary Disease; CRP:C-reactive protein; PCT: Procalcitonin; BNP: brain natriuretic peptide; PaO2: partial pressure of oxygen in artery; PaCO2: partial pressure of carbon dioxide in arterial blood; SaO2: oxygen saturation in arterial blood; ICS: inhaled corticosteroids

**Table 5:** The impact of smoking cessation on comorbidities and left heart function in early or late smoking COPD patients (after adjusting smoking index)

Variables	Early smoking (*n* = 104)	Late smoking (*n* = 104)	*P*-value
Echocardiography			
LVD, mm	44(41,47)	42(40,44)	0.003
LAS, mm	30(27,33)	29(27,33)	0.879
RVD, mm	29(27,32)	28(26,32)	0.360
RAS, mm	29(26,32)	28(27,31)	0.378
AO, mm	30(28,33)	30(27,32)	0.316
PA, mm	22(20,24)	22(21,25)	0.704
PA/AO	0.72(0.69,0.82)	0.77(0.69,0.88)	0.233
LVSD, %	9(8,10)	9(8,10)	0.813
LVPWD, mm	9(8,10)	9(8,10)	0.527
PAV, cm/s	80(71,97)	84(80,99)	0.141
EF, %	62(60,65)	61(60,64)	0.733
FS, %	33(30,35)	32(30,34)	0.646
Comorbidities and complications			
Coronary heart disease, %	11.5	21.2	0.061
Hypertension, %	27.9	44.4	0.057
Diabetes, %	13.5	8.7	0.269
Pneumonia, %	19.2	22.1	0.607
Bronchiectasis, %	12.5	14.4	0.685
Respiratory failure, %	40.4	41.7	0.842
Prior pulmonary TB, %	25.0	31.4	0.309
Cor pulmonale, %	28.8	27.9	0.878

**Notes**: LVD: Left Ventricular Diameter; LAS: Left Ventricular Diameter; RVD: Reft Ventricular Diameter; RAS: Left Ventricular Diameter; AO: Aortic Diameter; PA: Pulmonary Artery Diameter; LVSD: Left Ventricular Systolic Dysfunction; LVPWD: Left Ventricular Posterior Wall Diameter; PAV: Pulmonary Artery Velocity; EF: Ejection Fraction; FS: Fractional Shortening

**Table 6:** The impact of smoking cessation on short- and long-term prognosis in early or late smoking COPD patients (after adjusting smoking index)

Variables	Early smoking (*n* = 104)	Late smoking (*n* = 104)	*P*-value
Number of mild AEs one year after discharge, times	0(0,0)	0(0,0)	0.998
Number of AECOPDs one year after discharge, times	1(0,1)	1(0,2)	0.416
Incidence of death within one year after discharge(%)	12.5	6.7	0.158
Incidence of death within 3 years after discharge(%)	24.0	14.4	0.079
Incidence of death within long term after discharge(%)	32.7	17.3	0.010

**Notes**: AE: Acute Exacerbation; AECOPD: Acute Exacerbation of Chronic Obstructive Pulmonary Disease

## Discussion

Our study investigated the impact of early smoking, particularly during adolescent development stages (ADS), on the progression of COPD. We found that early smoking is associated with low cessation rates, high smoking indices, compromised lung function, and enlarged left ventricular diameter in COPD patients. These patients also displayed poorer three-year post-discharge and overall long-term survival rates. Alarmingly, even among those who quit smoking, early smokers had worse outcomes than those who started smoking later, suggesting damage in ADS might last even after cessation. Our research emphasizes the need for early and targeted interventions to mitigate the effects of smoking on this at-risk population.

We observed that early smoking COPD patients displayed a significantly higher smoking index, coupled with a lower likelihood of successfully quitting smoking. This intriguing observation aligns with prior research highlighting the association between adolescent smoking intensity and adult smoking prevalence and cessation rates [23]. However, the underlying mechanisms driving the persistence of smoking among individuals with early and intense smoking histories remain a subject of inquiry [24–26]. It is plausible that early exposure to nicotine plays a pivotal role, as suggested by a 2012 study emphasizing the heightened vulnerability of neurodevelopment to the effects of nicotine in individuals who initiated smoking early in life [27]. It is imperative to acknowledge that the current study did not encompass specific measures related to nicotine addiction or other potential explanatory factors, including deviant tendencies, behavioral and emotional disorders, as well as adult and peer smoking role models [28]. Our study also found early smoking COPD patients had significantly poorer lung function, which may be due to the cumulative effects of airway inflammation, alveolar destruction, and fibrotic effects caused by long-term smoking. To remove the bias from the heavier exposure to cigarette, the population was adjusted for smoking index, and still showed a worse condition and prognosis in early smoking COPD patients. These findings are consistent with a study published in the journal Thorax, which highlighted a strong relationship between early smoking and reduced lung function, which declines faster in early smokers [29]. The deleterious effects of early smoking on the lungs may be due to the fact that smoking-induced oxidative stress and inflammatory responses in the developing lung cause more lasting and widespread damage [30, 31].

Our data reveal that early smoking COPD patients suffer more severe damage to the heart, which is mainly reflected in the thickening of the left ventricular wall. Both active and passive smoking are capable of exacerbating the risk of atherosclerosis at various stages, a process that begins with endothelial dysfunction and may progress to a variety of cardiovascular diseases [21, 32, 33]. Further studies have also found that smoking behavior in young people is strongly associated with cardiovascular disease even after controlling for other risk factors [34]. Not only that, but smoking cessation has been shown to have a positive effect on improving endothelium-dependent vasodilatory function and reducing cardiovascular disease morbidity and mortality [31]. Thus, early smoking may interfere with normal cardiovascular development and promote the formation and progression of heart lesions by increasing the risk of atherosclerosis.

Our study suggests that early smoking COPD patients have lower survival rates, which may be related to irreversible cardiac and pulmonary developmental damage, which includes factors such as systemic inflammation and endocrine metabolic disorders [35, 36]. Nevertheless, smoking cessation may improve the prognosis of COPD patients by reducing the risk of cardiovascular disease and improving lung function and quality of life [16, 19, 37]. However, it is noteworthy that even among COPD patients who successfully quit smoking, individuals who started smoking early still showed worse health and survival outcomes compared to late smokers, especially with higher long-term mortality and shorter survival time.

Based on our findings, we advocate the inclusion of a detailed smoking history in routine clinical assessments. Clinicians should pay particular attention to the age of smoking initiation in COPD patients, as this may significantly influence treatment decisions and prognostic assessment. Treatment strategies must be tailored to the specific needs of this subgroup to improve their prognosis, such as more cardiac monitor and support. In addition, it is worth noting that widespread evidence suggests that trends and patterns in age of smoking initiation can be influenced by effective smoking intervention policies [38–40]. These policies have the potential to prevent smoking initiation or delay the onset of the habit. Our findings provide an empirical basis that should motivate policymakers, especially in areas with high smoking prevalence, to increase efforts to curb access to tobacco among the younger population, which should also be complemented by strong public awareness campaigns emphasizing the dangers of early smoking initiation.

While our study provides valuable insights, it has several limitations that warrant attention. As an observational cohort study, it may be subject to selection bias and the individuals participating in the study may not be representative of the entire target population, this bias may weaken the external validity of our findings and limit the ability to generalize the results to a wider population. Additionally, it is noteworthy that our study cohort predominantly consists of male participants, which introduces a further limitation regarding gender representation. Therefore our findings need to be further substantiated in large prospective randomized controlled trials.

## Conclusion

Early smoking COPD patients showed a number of unfavorable clinical characteristics including lower smoking cessation rates, higher smoking index, poorer lung function, thicker left ventricular diameter, more acute exacerbations in the past year and higher risk of death after three years and in the long term. In addition smoking initiation at the stage of alveolar development contributed to their poorer smoking cessation outcomes. The correlation between age of smoking initiation and prognosis of COPD allows healthcare professionals to prioritize early interventions by recognizing that patients with an earlier age of smoking initiation are more likely to develop severe COPD and tailoring smoking cessation strategies to them. In addition, these findings may help to develop targeted anti-smoking campaigns and policies to reduce the burden of COPD.

### Electronic supplementary material

Below is the link to the electronic supplementary material.

Supplementary Material 1

## Data Availability

No datasets were generated or analysed during the current study.
