# Supplementary material for: Early smoking lead to worse prognosis of COPD patients: a real world study
Source: Respir Res. 2024 Mar 25;25:140. doi: 10.1186/s12931-024-02760-y (PMC10964646; doi:10.1186/s12931-024-02760-y)
Supplement: Supplementary file 1 — Supplementary Material 1 [file 12931_2024_2760_MOESM1_ESM.docx]

**Supplementary Table legends**

**Supplementary Table 1** Baseline characteristics (before adjusting smoking index)

| **Variables** | | **Early smoking (n=384)** | | **Late smoking (n=313)** | | ***P*-value** | |
| --- | --- | --- | --- | --- | --- | --- | --- |
| Age, years | | 66(59,70) | | 72(66,78) | | <0.001 | |
| Male, % | | 379(98.7%) | | 301(96.2%) | | 0.031 | |
| BMI, kg/m^2^ | | 21.36(18.84,24.14) | | 21.26(18.80,24.45) | | 0.900 | |
| Smoking index, packets/year | | 50(40,75) | | 30(20, 50) | | <0.001 | |
| Spirometry(post-bronchodilation) |  | |  | |  | |  |
| FEV1 % predicted | | 29(21,39.68) | | 32(23.8,47) | | 0.006 | |
| FEV1/FVC, % | | 35.95(29,45.43) | | 38.61(31.09,50) | | 0.011 | |
| mMRC | | 3(2,4) | | 3(2,4) | | 0.334 | |
| CAT | | 24(18,28) | | 23(18,28) | | 0.447 | |
| Frequency of AEs in the last 12 months, times | | 2(1,3) | | 1(1,3) | | 0.031 | |
| Frequency of admission for AECOPD in the last 12 months, times | | 1(1,3) | | 2(1,3) | | 0.119 | |
| Laboratory investigations on admission |  | |  | |  | |  |
| WBC count, x 10^9^/L | | 7.25(5.68,9.48) | | 6.99(5.73,8.92) | | 0.436 | |
| Neutrophil count, x 10^9^/L | | 5.04(3.89,7.07) | | 5.10(3.94,6.92) | | 0.985 | |
| Eosinophil count, x 10^9^/L | | 0.15(0.07,0.24) | | 0.12(0.05,0.23) | | 0.114 | |
| CRP, mg/l | | 5.73(2.68,17.38) | | 8.87(3.27,23.38) | | 0.104 | |
| PCT, mg/l | | 0.05(0.05,0.12) | | 0.1(0.05,0.15) | | 0.126 | |
| BNP, pg/ml | | 140(50,632) | | 120(50,430) | | 0.741 | |
| PaCO_2_, mm/Hg | | 51(45,59.3) | | 49(42,57) | | 0.023 | |
| PaO_2_, mm/Hg | | 69(56,80) | | 69(57,78) | | 0.976 | |
| SaO_2_, % | | 93(89,96) | | 94(89,96) | | 0.133 | |
| ICS therapy during stable stage, % | | 75.5 | | 76.7 | | 0.722 | |
| Triple therapy during stable stage, % | | 30.5 | | 30.0 | | 0.901 | |

**Notes:** Date are presented as median(IQR) or n(%). FVC: forced vital capacity; FEV1: forced expiratory volume in 1 s; mMRC: modified Medical Research Council; CAT: COPD Assessment Test; TB: tuberculosis; AE: Acute Exacerbation; AECOPD: Acute Exacerbation of Chronic Obstructive Pulmonary Disease; CRP:C-reactive protein; PCT: Procalcitonin; BNP: brain natriuretic peptide; PaO2: partial pressure of oxygen in artery; PaCO2: partial pressure of carbon dioxide in arterial blood; SaO2: oxygen saturation in arterial blood; ICS: inhaled corticosteroids.

**Supplementary Table 2** The effect of early or late smoking on comorbidities and left heart function in COPD patients (before adjusting smoking index)

| **Variables** | | **Early smoking (n=384)** | | **Late smoking (n=313)** | | ***P*-value** | |
| --- | --- | --- | --- | --- | --- | --- | --- |
| Echocardiography | |  | |  | |  | |
| LVD, mm | | 44(40,47) | | 42(40,45) | | 0.016 | |
| LAS, mm | | 30(26.25,33) | | 30(27,33) | | 0.600 | |
| RVD, mm | | 29(26,32) | | 29(27,32) | | 0.478 | |
| RAS, mm | | 29(26,32) | | 29(27,32) | | 0.436 | |
| AO, mm | | 29(28,32) | | 30(27,32) | | 0.382 | |
| PA, mm | | 22(20,24) | | 22(20,25) | | 0.359 | |
| PA/AO | | 0.75(0.69,0.83) | | 0.75(0.69,0.88) | | 0.654 | |
| LVSD, % | | 9(8,10) | | 9(8,10) | | 0.243 | |
| LVPWD, mm | | 9(8,10) | | 9(8,10) | | 0.460 | |
| PAV, cm/s | | 90(80,100) | | 90(80,100) | | 0.235 | |
| EF, % | | 61(60,64) | | 60(60,62) | | 0.720 | |
| FS, % | | 32(30,34) | | 32(30,34) | | 0.635 | |
| Comorbidities and complications |  | |  | |  | |  |
| Coronary heart disease, % | | 13.8 | | 17.9 | | 0.139 | |
| Hypertension, % | | 30.7 | | 41.5 | | 0.003 | |
| Diabetes, % | | 10.9 | | 12.8 | | 0.453 | |
| Pneumonia, % | | 10.2 | | 17.6 | | 0.004 | |
| Bronchiectasis, % | | 11.2 | | 15.7 | | 0.084 | |
| Respiratory failure, % | | 39.6 | | 39.7 | | 0.966 | |
| Prior pulmonary TB, % | | 27.9 | | 35.4 | | 0.034 | |
| Cor pulmonale, % | | 22.9 | | 21.4 | | 0.633 | |

**Notes:** LVD: Left Ventricular Diameter; LAS: Left Ventricular Diameter; RVD: Reft Ventricular Diameter; RAS: Left Ventricular Diameter; AO: Aortic Diameter; PA: Pulmonary Artery Diameter; LVSD: Left Ventricular Systolic Dysfunction; LVPWD: Left Ventricular Posterior Wall Diameter; PAV: Pulmonary Artery Velocity; EF: Ejection Fraction; FS: Fractional Shortening.

**Supplementary Table 3** The effect of early or late smoking on prognosis in COPD patients (before adjusting smoking index)

| **Variables** | **Early smoking (n=384)** | **Late smoking (n=313)** | ***P*-value** |
| --- | --- | --- | --- |
| Number of mild AEs one year after discharge, times | 0(0,0) | 0(0,0) | 0.904 |
| Number of AECOPDs one year after discharge, times | 1(0,2) | 1(0,2) | 0.660 |
| Incidence of death within one year after discharge(%) | 11.2 | 10.2 | 0.680 |
| Incidence of death within 3 years after discharge(%) | 22.4 | 24.6 | 0.494 |
| Incidence of death more than 3 years after discharge(%) | 25.8 | 30.4 | 0.181 |

**Notes:** AE: Acute Exacerbation; AECOPD: Acute Exacerbation of Chronic Obstructive Pulmonary Disease.

**Supplementary Table 4** Early smoking impaired smoking cessation (before adjusting smoking index)

| **Variables** | **Early smoking (n=206)** | **Late smoking (n=253)** | ***P*-value** |
| --- | --- | --- | --- |
| Age, years | 66(59,70) | 72(66, 78) | <0.001 |
| Male, % | 99.5 | 96.4 | 0.025 |
| BMI, kg/m^2^ | 21.60(19.38,24.05) | 21.46(19.04,24.64) | 0.940 |
| Smoking index ,packets/year | 50(40,68) | 30(20,40) | <0.001 |
| Duration of smoking cessation, years | 2(1,5) | 10(3, 20) | <0.001 |
| Spirometry(post-bronchodilation) |  |  |  |
| FEV1 % predicted | 27.9(20.7,37.8) | 32(22.7,45.8) | 0.007 |
| FEV1/FVC, % | 35(28.3,42.8) | 38(30.6,49.7) | 0.017 |
| mMRC | 3(2,4) | 3(2,4) | 0.440 |
| CAT | 23(18,28) | 24(19,28) | 0.636 |
| Frequency of AEs in the last 12 months, times | 2(1,3) | 2(1,3) | 0.436 |
| Frequency of admission for AECOPD in the last 12 months, times | 2(1,3) | 1(1,2) | 0.835 |
| Laboratory investigations on admission |  |  |  |
| WBC count, x 10^9^/L | 7.54(6.04,9.85) | 6.83(5.47,8.99) | 0.023 |
| Neutrophil count, x 10^9^/L | 5.45(4.05,7.97) | 5.13(3.92,6.82) | 0.216 |
| Eosinophil count, x 10^9^/L | 0.13(0.05,0.24) | 0.11(0.05,0.22) | 0.159 |
| CRP, mg/l | 9.97(3.47,30.85) | 9.20(3.69,24.55) | 0.441 |
| PCT, mg/l | 0.05(0.05,0.12) | 0.10(0.05,0.14) | 0.269 |
| BNP, pg/ml | 120(50,402) | 120(50,382) | 0.615 |
| PaCO_2_, mm/Hg | 50(43,60) | 49(42,59) | 0.217 |
| PaO_2_, mm/Hg | 69(56,82) | 67(54,78) | 0.188 |
| SaO_2_, % | 94(88,96) | 93(88,95) | 0.484 |
| ICS therapy during stable stage, % | 7.54(6.04,9.85) | 74.7 | 0.916 |
| Triple therapy during stable stage, % | 5.45(4.05,7.97) | 27.3 | 0.890 |

**Notes:** Date are presented as median(IQR) or n(%). FVC: forced vital capacity; FEV1: forced expiratory volume in 1 s; mMRC: modified Medical Research Council; CAT: COPD Assessment Test; TB: tuberculosis; AE: Acute Exacerbation; AECOPD: Acute Exacerbation of Chronic Obstructive Pulmonary Disease; CRP:C-reactive protein; PCT: Procalcitonin; BNP: brain natriuretic peptide; PaO2: partial pressure of oxygen in artery; PaCO2: partial pressure of carbon dioxide in arterial blood; SaO2: oxygen saturation in arterial blood; ICS: inhaled corticosteroids.

**Supplementary Table 5** The impact of smoking cessation on comorbidities and left heart function in early or late smoking COPD patients (before adjusting smoking index)

| **Variables** | | **Early smoking (n=206)** | | **Late smoking (n=253)** | | ***P*-value** | |
| --- | --- | --- | --- | --- | --- | --- | --- |
| Echocardiography | |  | |  | |  | |
| LVD, mm | | 44(41,47) | | 42(40,45) | | 0.025 | |
| LAS, mm | | 29(27,33) | | 30(27,33) | | 0.442 | |
| RVD, mm | | 29(26,32) | | 29(26,32) | | 0.881 | |
| RAS, mm | | 29(26,32) | | 29(27,32) | | 0.907 | |
| AO, mm | | 30(28,32) | | 30(27,33) | | 0.853 | |
| PA, mm | | 22(20,24) | | 22(20,25) | | 0.610 | |
| PA/AO | | 0.74(0.69,0.84) | | 0.75(0.68,0.88) | | 0.568 | |
| LVSD, % | | 9(8,10) | | 9(8,10) | | 0.327 | |
| LVPWD, mm | | 9(8,10) | | 9(8,10) | | 0.506 | |
| PAV, cm/s | | 80(80,94) | | 80(80,90) | | 0.765 | |
| EF, % | | 60(60,64) | | 60(60,62) | | 0.946 | |
| FS, % | | 32(30,35) | | 32(30,34) | | 0.796 | |
| Comorbidities and complications |  | |  | |  | |  |
| Coronary heart disease, % | | 15.5 | | 17.0 | | 0.673 | |
| Hypertension, % | | 26.7 | | 40.3 | | 0.002 | |
| Diabetes, % | | 12.1 | | 11.1 | | 0.722 | |
| Pneumonia, % | | 15.0 | | 18.2 | | 0.372 | |
| Bronchiectasis, % | | 11.7 | | 15.8 | | 0.201 | |
| Respiratory failure, % | | 40.3 | | 40.9 | | 0.900 | |
| Prior pulmonary TB, % | | 27.7 | | 35.1 | | 0.091 | |
| Cor pulmonale, % | | 27.7 | | 22.5 | | 0.205 | |

**Notes:** LVD: Left Ventricular Diameter; LAS: Left Ventricular Diameter; RVD: Reft Ventricular Diameter; RAS: Left Ventricular Diameter; AO: Aortic Diameter; PA: Pulmonary Artery Diameter; LVSD: Left Ventricular Systolic Dysfunction; LVPWD: Left Ventricular Posterior Wall Diameter; PAV: Pulmonary Artery Velocity; EF: Ejection Fraction; FS: Fractional Shortening.

**Supplementary Table 6** The impact of smoking cessation on short- and long-term prognosis in early or late smoking COPD patients (before adjusting smoking index)

| **Variables** | **Early smoking (n=206)** | **Late smoking (n=253)** | ***P*-value** |
| --- | --- | --- | --- |
| Number of mild AEs one year after discharge, times | 0(0,0) | 0(0,0) | 0.793 |
| Number of AECOPDs one year after discharge, times | 0(0,2) | 1(0,2) | 0.127 |
| Incidence of death within one year after discharge(%) | 12.6 | 11.9 | 0.804 |
| Incidence of death within 3 years after discharge(%) | 25.2 | 26.1 | 0.837 |
| Incidence of death within long term after discharge(%) | 27.2 | 32.4 | 0.225 |

**Notes:** AE: Acute Exacerbation; AECOPD: Acute Exacerbation of Chronic Obstructive Pulmonary Disease.
